# Supplementary material for: Genetic comparisons of interleukin-17 reveal a framework for complex signaling evolution
Source: bioRxiv. 2026 Apr 14:2026.04.13.718218. Preprint. [Version 1] doi: 10.64898/2026.04.13.718218 (PMC13105070; doi:10.64898/2026.04.13.718218)
Supplement: Supplement 3 [file media-3.zip › Supplemental files for paper/ERC/IL17xIL17_comparisons/IL17RD_IL17RA.pdf]

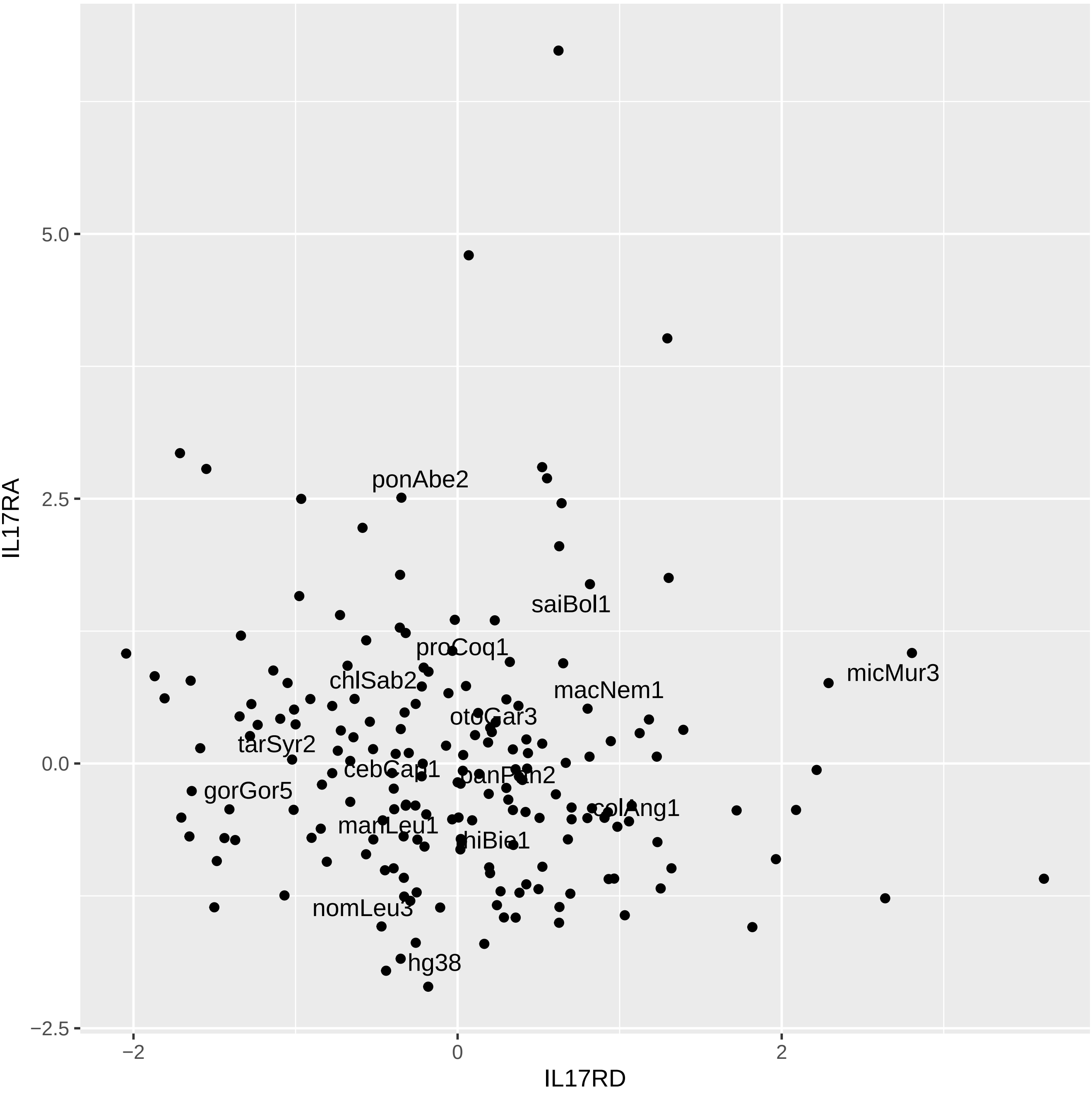

IL17RA
IL17RD
5.0
2.5
0.0
−2.5
−2
0
2
ponAbe2
saiBol1
proCoq1
micMur3
chlSab2
macNem1
otoGar3
tarSyr2
cebCap1
panPan2
gorGor5
colAng1
manLeu1
nomLeu3
hg38
